# One-lung ventilation in a patient with Fontan circulation undergoing diaphragmatic plication surgery: a case report

**DOI:** 10.1186/s40981-019-0270-x

**Published:** 2019-08-01

**Authors:** Yusuke Sasaki, Jungo Kato, Rie Minoshima, Hiromasa Nagata, Shizuka Minamishima, Takeshi Suzuki, Hiroshi Morisaki

**Affiliations:** Department of Anesthesiology, Keio University School of Medicine, 35 Shinanomachi, Shinjuku-ku, Tokyo, 1608582 Japan

**Keywords:** One lung ventilation, Fontan circulation, Hypoxic pulmonary vasoconstriction, Pulmonary angiography, Hemidiaphragmatic paralysis

## Abstract

**Background:**

Surgeries requiring one-lung ventilation (OLV) in patients with Fontan circulation pose great challenges. However, little information is available regarding the safety of OLV in Fontan patients when hemidiaphragmatic paralysis is present.

**Case presentation:**

A 41-year-old woman who underwent repeated Fontan procedures was re-admitted to our hospital because of worsening shortness of breath. As left hemidiaphragmatic paralysis was considered to be contributing to her symptom, an open thoracic left diaphragmatic plication surgery was scheduled. A preoperative pulmonary artery angiogram revealed a remarkably reduced blood flow to the left lung. The surgeon requested OLV during the surgery. Despite our concern regarding the impact of OLV on the Fontan circulation, OLV did not result in major hemodynamic changes.

**Conclusion:**

OLV can be safely implemented in patients with hemidiaphragmatic paralysis with preserved Fontan circulation. Preoperative pulmonary artery angiography may provide useful information for estimating the impact of OLV on the Fontan circulation.

## Background

The Fontan procedure is a well-established surgical treatment for single ventricle type of congenital cardiac defects [1]. As the survival rate following the Fontan procedure has improved, the chances of patients with Fontan circulation undergoing non-cardiac surgeries are increasing [2, 3].

While general anesthesia itself is considered to be a high-risk intervention for patients with Fontan circulation [4, 5], surgeries requiring one-lung ventilation (OLV) pose further challenges for anesthesiologists. Hemidiaphragmatic paralysis is one of the major complications associated with the Fontan procedure that may require surgical intervention [6], for which OLV would be advantageous during operation.

Given the absence of a functional pumping ventricle in patients with Fontan circulation, it is crucial to keep the pulmonary vascular resistance (PVR) low. However, OLV can potentially raise the PVR by several mechanisms [7, 8]. Lending support to such concerns, previous case reports have documented disruption of Fontan circulation during OLV in patients undergoing thoracic surgeries [9, 10]. On the other hand, little information is available regarding the safety of OLV in Fontan patients specifically when hemidiaphragmatic paralysis is present.

Herein, we report a case of a Fontan patient with hemidiaphragmatic paralysis who underwent left diaphragm plication surgery under OLV. Our experience suggests that preoperative evaluation of the pulmonary blood flow distribution may be beneficial in estimating the impact of OLV on the hemodynamics in Fontan patients.

## Case presentation

**Fig. 1:**
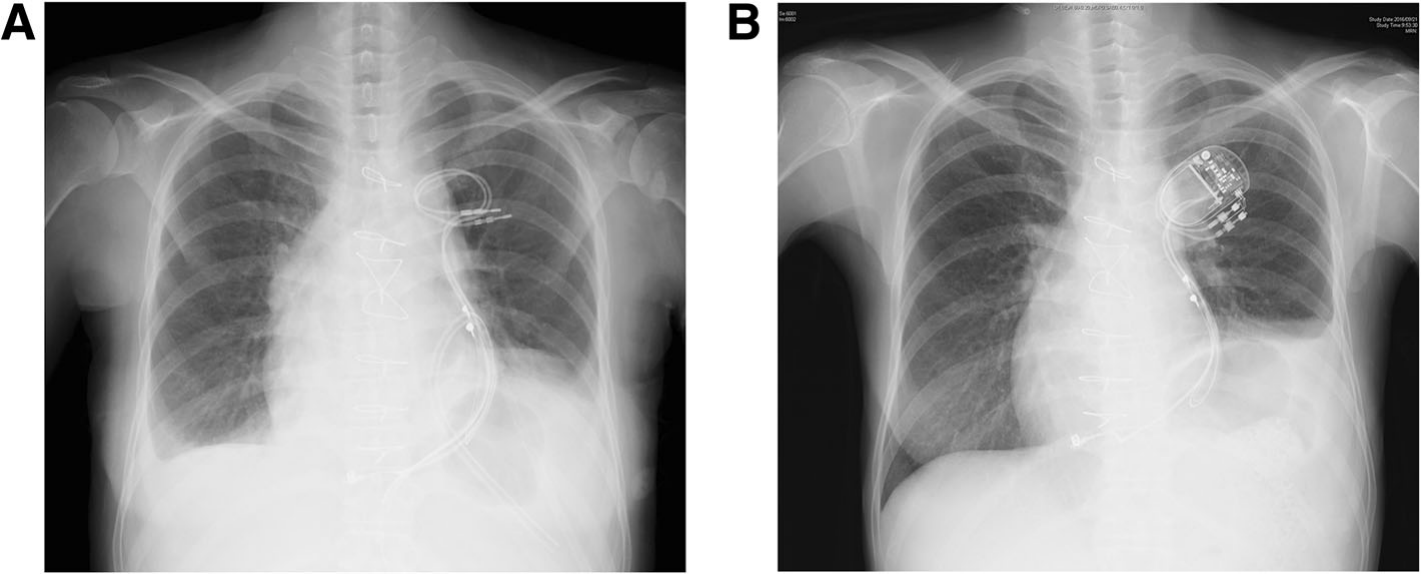
The chest X-ray images **a** immediately after the TCPC conversion on the fourth postoperative day and **b** on the current admission, showing significant elevation of the left dome of the diaphragm, suggesting left hemidiaphragmatic paralysis

A 41-year-old woman was re-admitted to our hospital with a history of persistent symptoms of heart failure, including shortness of breath. In her past medical history, she had been diagnosed at birth to have a single ventricle type of cardiac defect, with a hypoplastic right ventricle. She underwent an atriopulmonary connection (APC)-type Fontan procedure at the age of 9 years. Subsequently, due to worsening of the heart failure and new onset of atrial flutter, she underwent total cavopulmonary connection (TCPC) conversion and pacemaker placement surgery at the age of 41 years, 5 months prior to her current re-admission. The chest X-ray image immediately after surgery revealed a significantly elevated left dome of the diaphragm, consistent with a left hemidiaphragmatic paralysis (Fig. 1a). While she had been mostly asymptomatic for 2 months following the TCPC conversion, the suspension of diuretics and anemia triggered by menorrhea worsened her heart failure, which manifested as lower extremity edema and ascites. The nadir of her hemoglobin level was 6.5 g/dL. Her B-type natriuretic peptide (BNP) value gradually increased from 40.4 to 111.8 pg/mL during this period. After the resumption of diuretics and 2 units of red blood cell transfusion, her heart failure entered remission with some fluctuations in her symptom.

**Fig. 2:**
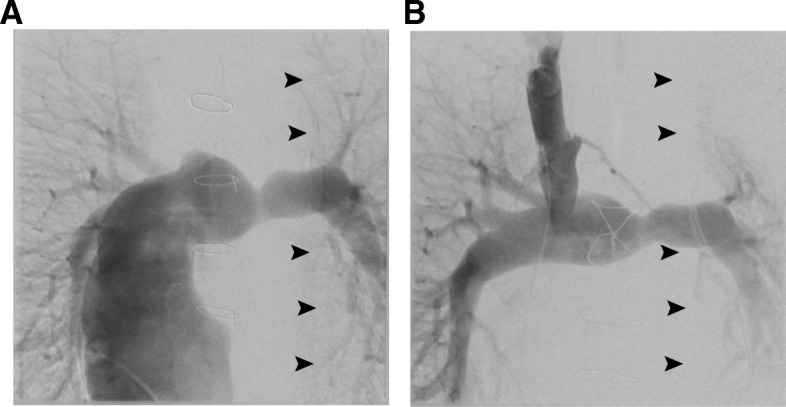
Pulmonary angiograms of **a** before and **b** after the TCPC conversion showing reduced blood distribution to left lung due to the left hemidiaphragmatic paralysis following the TCPC conversion surgery (arrowheads)

The chest X-ray image on the current admission showed a further reduction of left lung volume compared to the one immediately after the TCPC conversion surgery (Fig. 1b). Transthoracic echocardiography (TTE) showed preserved ventricular systolic function with a fractional area change of 46.4% and mild mitral valve regurgitation. The preoperative laboratory data showed a BNP level of 62.5 pg/mL. A cardiac catheterization study revealed increased filling pressures in SVC, IVC, right and left pulmonary arteries, and in the conduit, as compared to the values measured before the TCPC conversion surgery. Consistent with the above, the pulmonary vascular resistance was also significantly elevated (Table 1). A pulmonary artery angiogram showed remarkably reduced blood flow to the left lung as compared to that before the TCPC conversion (Fig. 2).

**Table 1:** Preoperative cardiac catheter laboratory findings

	Before TCPC conversion	Immediately after TCPC conversion	Before current surgery
SVC/IVC/conduit (mmHg)	9/9/9	16^a^	16/16/16
lt. PA/rt. PA (mmHg)	9/9		14/14
lt. PC wedge (mmHg)	7		11
rt. PC wedge (mmHg)	5		10
PVR (w.u.)	0.4		1.4

*TCPC* total cavopulmonary connection, *SVC* superior vena cava, *IVC* inferior vena cava, *PA* pulmonary artery, *PC* pulmonary capillary, *PVR* pulmonary vascular resistance

^a^Average central venous pressure (CVP) measured at the bedside through postoperative day 1–3

The reduced left lung volume due to the left hemidiaphragmatic paralysis, plausibly caused by unilateral phrenic nerve damage during the previous surgery, was suspected as being the precipitating factor of her symptom, and therefore, a left diaphragmatic plication surgery was scheduled. Due to the concern of intra-thoracic adhesion because of multiple previous operations, the open thoracic approach was chosen. For better exposure of the surgical field, the surgeon requested OLV during the surgery. Preoperative blood examination did not reveal any significant abnormality except for a reduced platelet count (8.2 × 10^4^/μl). Although the preoperative platelet count was slightly lower than the institutional limit for neuraxial anesthesia (10 × 10^4^/μL), we decided to perform epidural anesthesia, taking into account its beneficial effects on the respiratory function during the postoperative period.

The epidural catheter was placed preoperatively at the epidural space at Th6/7. The general anesthesia was induced with a propofol target-controlled infusion (TCI) set at 2.5 μg/mL and fentanyl (100 μg) and was maintained with propofol TCI. A left-sided double-lumen tube (35 Fr. Broncho-cath, Covidien) was placed for the OLV. To minimize the adverse effects of mechanical ventilation on the PVR, the ventilator was set in the pressure control mode with an FiO_2_ of 1.0, peak inspiratory pressure of 22 cm H_2_O at an inspiration:expiration ratio of 1:2.6, and a positive end-expiratory pressure (PEEP) of 5 cm H_2_O. Intraoperative analgesia was achieved with remifentanil infusion and intermittent epidural injection of levobupivacaine. Muscle relaxation was achieved with intermittent rocuronium injections. To prepare for hemodynamic deterioration as a result of OLV, the arterial line at the right radial artery and central venous line at the right internal jugular vein were secured. A transesophageal echocardiography (TEE) probe was also placed. The pacing rate was set at 70 beats/min in the DOO mode. In case of circulatory collapse during the surgery, a cardiopulmonary bypass (CPB) machine and an inhaled nitric oxide (iNO) delivery system, as well as inotropes and vasopressors, were placed on standby for emergent use.

**Fig. 3:**
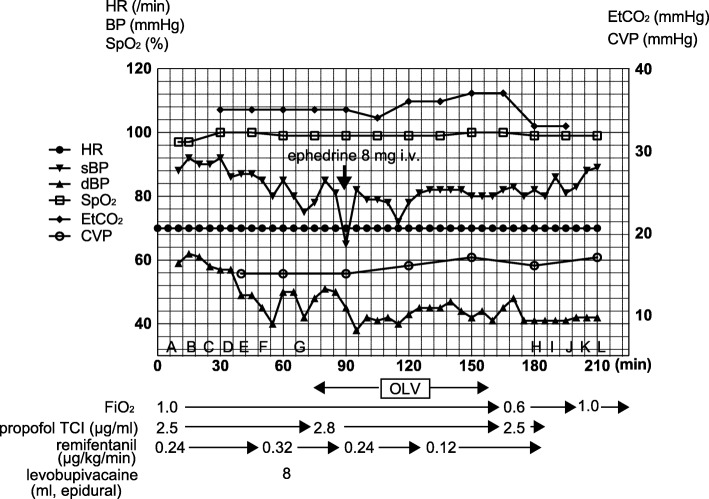
Intraoperative hemodynamic and respiratory changes during the diaphragmatic plication surgery: there was no major hemodynamic deterioration associated with the implementation of one-lung ventilation (OLV). **a** Epidural catheter placement, **b** start of general anesthesia, **c** intubation, **d** central line placement, **e** TEE insertion, **f** rt. decubitus position, **g** start of operation, **h** end of operation, **i** supine position, **j** TEE removal, **k** extubation, and **l** end of anesthesia

The open thoracic left diaphragm plication surgery was initiated with the patient placed in the right decubitus position. Following the opening of the thoracic cavity, collapse of the lung on the surgical side was successfully achieved. Despite our concern about hemodynamic deterioration, the OLV did not result in any major changes of the central venous pressure, blood pressure, or arterial oxygen saturation (Fig. 3). TEE did not show any remarkable changes in the ventricular function either.

**Fig. 4:**
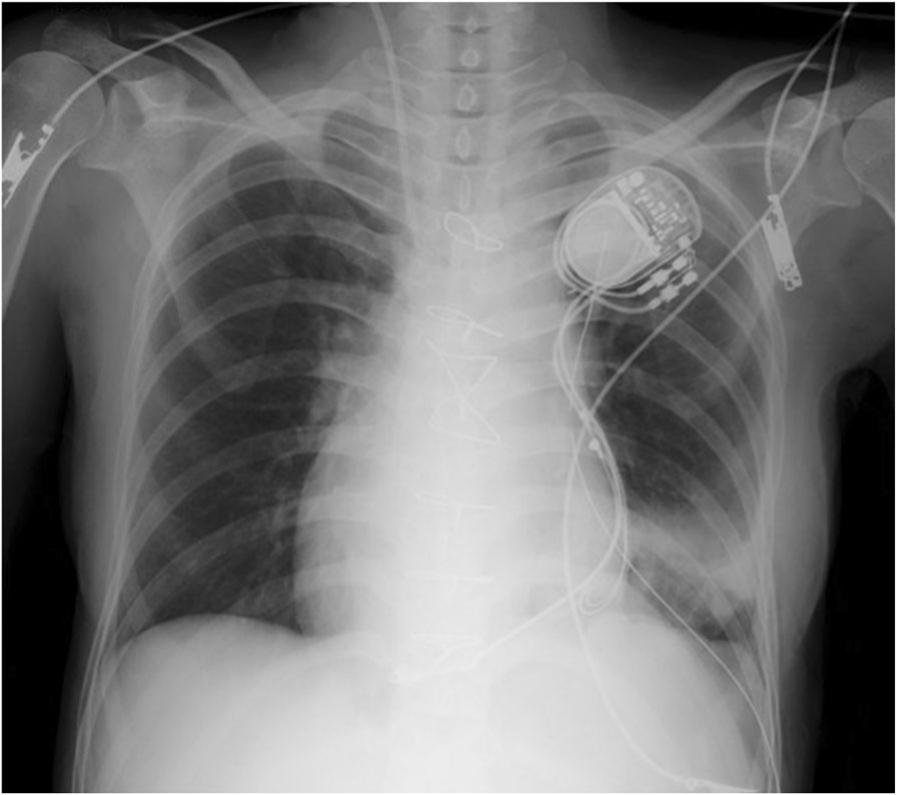
The postoperative chest X-ray after the extubation showing the successful re-expansion of the left lung

The total OLV time was 85 min, and the patient remained hemodynamically stable throughout this period, except for one episode of hypotension that was easily managed a bolus injection of ephedrine. The surgery was completed within 2 h and the patient was successfully extubated in the operating room. The postoperative chest X-ray showed the successful re-expansion of the left lung (Fig. 4).

Her postoperative pain was successfully managed by patient-controlled epidural analgesia. Her postoperative course was uneventful, with improvement of the heart failure symptoms, and she was discharged on postoperative day 23.

## Discussion

Theoretically, OLV has several detrimental effects in patients with Fontan circulation. The physiological changes associated with OLV, such as hypercarbia, hypoxia, and hypoxic pulmonary vasoconstriction (HPV), disturb the blood flow to the pulmonary circulation through elevation of the PVR [7, 8, 11, 12]. Moreover, the rise in the intrathoracic pressure due to the high positive pressure ventilation during OLV also potentially interferes with the systemic venous return in patients with Fontan circulation [12]. In previous case reports, OLV indeed resulted in the disruption of Fontan circulation, manifesting as severe hypotension, elevated CVP, and elevated PVR, necessitating the use of vasopressors and inotropes [9, 10].

Contrary to these precedents, however, we did not encounter any major difficulties in the management of OLV in this patient. Given the limited number of available reports [9, 10, 13], it remains difficult to predict in which patients with Fontan circulation OLV would carry a higher risk of hemodynamic instability.

In the previous cases of OLV-induced hemodynamic derangements, the patients had severely failing Fontan circulation, manifesting as circulatory shock complicated by the presence of refractory atrial fibrillation and severe protein-losing enteropathy [9], or pre-existing oxygen desaturation [2]. Although the presently reported patient showed early signs of failing Fontan circulation preoperatively, she had not yet developed severe circulatory collapse or other Fontan-associated late comorbidities. In addition, as failing Fontan circulation can be characterized by an increased PVR [14], the relatively low preoperative PVR value, as well as the low BNP and the CVP that was equivalent to that immediately after TCPC conversion, may have indicated the existence of some reserve capacity in her Fontan circulation. Although further studies are mandatory, the presence of a reserve capacity in the Fontan circulation may partly determine the ability to tolerate OLV-induced hemodynamic derangements.

Second, the presence of the hemidiaphragmatic paralysis could have conferred a unique condition with respect to the impact of OLV on hemodynamics in Fontan circulation. In the presence of hemidiaphragmatic paralysis, HPV may have already been established to some extent preoperatively, owing to the reduced ipsilateral lung volume. This possibility is supported by the preoperative PA angiogram, which clearly showed a marked reduction of the blood flow to the left PA and compensatory increase of the right PA flow. Thus, the impact of further HPV induced by OLV on the re-distribution of the pulmonary blood flow may have been limited to be minimal.

The positioning of the patient during surgery also requires special considerations for the maintenance of Fontan circulation. Although the gravitational blood shift to the dependent lung during the lateral decubitus position would be favorable in terms of V/Q matching during OLV, this would also cause a further volume overload to the vasculature in the dependent lung. In addition, the lateral decubitus position is often associated with the development of atelectasis in the dependent lung, which can deleteriously increase the shunt fraction and PVR. Therefore, as we applied PEEP in the current case, strategies to prevent atelectasis during OLV are also important.

If the risk of hemodynamic derangement is estimated to be high, transabdominal approach for diaphragm plication can be a preferred option to circumvent OLV [15]. Care must be taken that a high degree of intra-thoracic adhesion due to repeated operations may complicate the procedure.

Taken together, our experience suggests that OLV can be safely implemented in patients with hemidiaphragmatic paralysis with preserved Fontan circulation. Thorough preoperative evaluation of the pulmonary blood flow pattern may provide useful information for estimating the impact of OLV on the hemodynamics in patients with Fontan circulation. Nevertheless, OLV in patients with Fontan circulation remains challenging and requires meticulous preoperative evaluation, preparation, and perioperative monitoring. Further accumulation of experience and integration of information are needed to establish the safe management of OLV in patients with Fontan circulation.

## Data Availability

The data in this case report are available from the corresponding author on reasonable requests.

## Abbreviations

APC: Atriopulmonary connection
BNP: B-type natriuretic peptide
CVP: Central venous pressure
HPV: Hypoxic pulmonary vasoconstriction
IVC: Inferior vena cava
OLV: One-lung ventilation
PA: Pulmonary artery
PVR: Pulmonary vascular resistance
SVC: Superior vena cava
TCI: Target-controlled infusion
TCPC: Total cavopulmonary connection
TEE: Transesophageal echocardiography
TTE: Transthoracic echocardiography
V/Q: Ventilation-to-perfusion ratio
